# Taxonomic assessment of *Allium* species from Kazakhstan based on ITS and *matK* markers

**DOI:** 10.1186/s12870-017-1194-0

**Published:** 2017-12-28

**Authors:** Saule Abugalieva, Lyubov Volkova, Yuliya Genievskaya, Anna Ivaschenko, Yuri Kotukhov, Gulzhahan Sakauova, Yerlan Turuspekov

**Affiliations:** 1 Institute of Plant Biology and Biotechnology, Almaty, Kazakhstan; 2 Ile-Alatau State National Nature Park, Almaty, Kazakhstan; 3 Altai Botanical Garden, Ridder, Kazakhstan; 4 Karatau State Nature Reserve, Kentau, Kazakhstan

**Keywords:** *Allium* taxonomy, ITS, *matK*, DNA barcoding, Subgenus *Reticulatobulbosa*

## Abstract

**Background:**

As part of nation-wide project to infer the genetic variation of the native flora in Kazakhstan, a study was attempted to assess phylogenetic relationships of endemic and rare *Allium* species. In total, 20 *Allium* species were collected in field trips in five different regions of Kazakhstan during 2015–2016. Most species (9) were collected in the southern part of the country along of Karatau mountains, followed by Altai mountains (5) in eastern Kazakhstan. The ITS and *mat*K DNA regions were applied in order to assess the taxonomic relationships among species. The major goal of the study was to assess the taxonomic position of five endemic and rare species from *Allium* subgenus *Reticulatobulbosa* collected in Karatau mountains of Southern Kazakhstan.

**Results:**

The 20 collected *Allium* species were assessed using morphological traits and a DNA barcoding approach. The morphological analyses of four different species in subgenus *Reticulatobulbosa* inferred similarities of *A. inconspicuum* and *A. barszchewskii* (both from section *Companulata*) that were separated from *A. oreoscordum* and *A. oreoprasoides* (section *Nigrimontana*) by several traits, including form of bulbs and leaves, presence of bracts, shape of perianth lobes and style. The Neighbor-Joining method was applied to generate ITS and *mat*K phylogenetic trees for two groups of populations: 1) 20 *Allium* species collected within the project, and 2) 50 *Allium* worldwide species.

**Conclusions:**

The analyses of nucleotide sequences of ITS and *mat*K robustly confirmed the monophyletic origin of the *Allium* species. The variability in 20 local *Allium* species in ITS was 6.6 higher than in *mat*K, therefore the topology of the ITS tree was better resolved. The taxonomy of *Allium* species largely coincided with a recent classification of this genus. Analyses of both ITS and *mat*K suggest that *A. oreoscordum* is genetically close to *A. oreoprasoides* in section *Nigrimontana* of subgenus *Reticulatobulbosa*. This result was also confirmed using morphological description of individual plants of four species in subgenus *Reticulatobulbosa*. The study is another contribution to taxonomy clarification in *Allium*.

**Electronic supplementary material:**

The online version of this article (10.1186/s12870-017-1194-0) contains supplementary material, which is available to authorized users.

## Background

*Allium* is one of the largest widespread genus in the Northern Hemisphere and it consists of more than 850 species [1]. The taxonomy of the *Allium* is well described in a number of classical botanical reviews [2, 3] and molecular genetics studies [1, 4]. Several initial publications based on DNA markers suggested polyphyletic origin of studied subgenera [5–7]. However, Friesen and co-authors [8] criticized this conclusion and noted the omission of species in analyzed subgenera and questioned the quality of studied materials in these publications. In the same report, Friesen et al. [8] proposed a new classification of *Allium* based on using rDNA ITS (internally transcribes spacers) sequences. The study suggested that *Allium* has a monophyletic origin and consists of three evolutionary lines with 15 subgenera and 72 sections. This classification was supported in several later studies [9–12] and became well adopted among *Allium* taxonomists. In spite of these substantial efforts towards understanding of evolutionary processes and taxonomy of the genus there are still many poorly described *Allium* taxa available in different parts of the World and active description of wild onion species is an ongoing process [1, 13, 14].

The Central Asian region, including Kazakhstan, is one of the important regions to study the evolution and distribution of *Allium* species. Kazakhstan is the largest Central Asian republic with total area of 2.7 million km^2^, which is ranking it as ninths largest country in the World by territory. The country is land locked and has rather unique flora consisting of about 6000 species with approximately 10% of them endemic to this region. Despite richness and uniqueness of the local flora and enormous volume of botanical studies, the plant community is still poorly characterized by using modern DNA based studies. There are only few examples in the literature suggesting taxonomy evaluation of wild plant species from this country, including in assessment of genetic variation in annual [15] and perennial species [16, 17]. This trend is slowly changing due to the launch of a new nation-wide research program [18] that combine efforts of local botanists and geneticists from Biotechnology Research Organizations, Botanical Gardens, State Nature Parks and Reserves. One of the examples of this trend is this collaborative study on reassessment of the taxonomy of *Allium* species growing in this region. According to Abdulina [19] there are 120 *Allium* species growing in different parts of the country. One of the regional hot spots of *Allium* diversity in Kazakhstan is the Karatau State Natural Reserve located in the southern part of Kazakhstan. Therefore, endemic, rare and economically important species of *Allium* in Karatau were a particular target in this study with a major focus on species in subg. *Reticulatobulbosa*.

Currently DNA barcoding tools [20] have been considered as one of the most informative and efficient approaches in evaluation of plant phylogeny, and successfully used in the molecular taxonomy of *Allium* [5]. The approach is based on alignment of short sequences of universal DNA markers from the nuclear and plastid genomes [21–23], and was applied in this study as well. The scope of the study was to assess of distribution areas of growth for endemic and rare *Allium* species in Kazakhstan, describe morphological patterns of poorly studied species, and clarify phylogenetic relationship of native species using a DNA barcoding approach. In particularly, we present the results of botanical and molecular phylogeny analysis of species in subg. *Reticulatobulbosa* native to Karatau State Nature Reserve in Southern Kazakhstan.

## Results

### Collecting *Allium* species in different regions of Kazakhstan

**Fig. 1:**
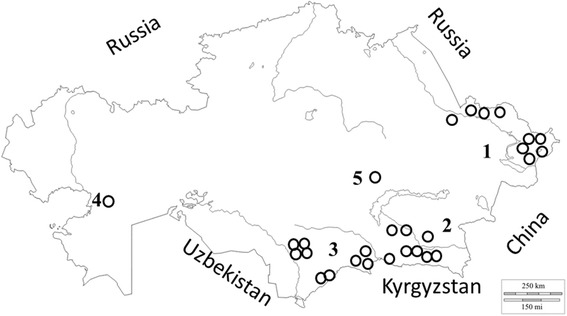
Locations of the collecting sites (open circles) in five different regions of Kazakhstan – East, South-east, South, West, and Center. Arabic figures reflect regions of collecting sites – East, South-east, South, West, and Center of Kazakhstan)

During 2015–2016 several collecting efforts in five regions of the country were conducted with the goal to sample endemic and rare *Allium* species. The summary of collecting trips is given in Table 1 and collecting areas are shown in Fig. 1. The area of sampling was stretched from Altai mountains in the east of the country to Ustyurt Plateau in the west. The highest number of *Allium* species was collected in southern Kazakhstan (9), including the mountainous area of Karatau State Nature Reserve. The geography of sampling areas varied from flat regions to mountainous areas and ranged from 96 m above sea level in Western Kazakhstan (*A. caspium*) to 2669 m in southern Kazakhstan (*A. caricoides*, Additional file 1), although within this project only a portion of the available wild onion species of the country were collected.

**Table 1:** Morphological description of four *Allium* species in subgenus *Reticulatobulbosa*

Species	*A.oreoprasoides*	*A.oreoscordum*	*A.inconspicuum*	*A. barszcewskii*
Section	*Nigrimontana*	Unknown	*Companulata*	*Companulata*
Subgenus	*Reticulatobulbosa*	*Reticulatobulbosa*	*Reticulatobulbosa*	*Reticulatobulbosa*
Traits:				
Bulb	Cylindrical-conical, 0,5-1 cm	Narrow conical, up to 1 cm wide	Oblong, ovoid-like, 0,5–1,5 cm wide	Conical, ovoid-like, 0,7–1,5 cm wide
Tunics	Reddish-brown, reticulate	Brownish, reticulate	Brown, reticulate	Brown, reticulate
Stem Length	20-30 cm, furrowed	25-50 cm	15-30 cm, thin	20-60 cm
Leaves (Number; Width)	Linear, flat (4–6; 2-3 mm)	Linear, flat (5–7; 3-5 mm, hard, shorter than stems)	Narrow-linear, filamentous, furrowed (1–2; 0,5-1 mm in width, a little shorter than stems)	Narrow-linear, smooth, furrowed (1; 1-3 mm, shorter than stems)
Spathe	With pointy end (1,5–2 times shorter than umbela)	Shortly-pointed (Equal to umbela)	Shortly-pointed (3 times shorter than umbela)	Shortly-pointed (2–3 times shorter than umbela)
Umbela	Hemispherical or spherical/multiflorous, dense	Spherical / large, dense	Bundle-like / non-multiflorous, loose	Bundle-like hemispherical / multiflorous, dense
Peduncle	Equal to each other, 2–3 times longer than perianth	Almost equal to each other, 1,5–3 times longer than perianth	Almost equal to each other, shorter, equal or longer than perianth	Unequal to each other, 2–3 times longer than perianth
Bracts	Present	Present	No	No
Perianth lobes (Length; Outter)	Pale pink with purple vein (4–5 mm; Boat-shaped)	Greenish-white on the back (4 mm; Boat-shaped)	Pale dirty-violet, darker on the back (8–11 mm; Linear-lanceolate)	Pink-violet, pink, white (7–14 mm; Lanceolate)
Stamina filaments	0.25 times longer than perianth, fused with each other and with perianth	1,5 times longer than perianth, fused with each other and with perianth	2 times shorter than perianth, fused with each other	1,5 times shorter than perianth, fused with each other and with perianth on 1/3 – 1/2
Style	Significantly extends out of corolla	Extends out of corolla	Does not extend out of corolla	Does not extend out of corolla

Evaluation of published *Allium* related literature suggested that species in sections *Nigrimontana* and *Campanulata* in subgen. *Reticulatobulbosa* are native to Karatau mountains in southern Kazakhstan and previously were poorly studied. Particularly, *A. oreoscordum* was not previously classified to any existing sections of *Reticulatobulbosa* and not mentioned by Friesen et al. [8]. The description of three species in Table 1 suggested they have similarities with *A. oreoscordum* in a number of traits, including peduncle length and samina filaments. At the same time, size of spathe and color of perianth lobes differentiated *A. oreoscordum* from the other three species. Also, traits such as form of bulbs and leaves, presence of bracts, shape of perianth lobes and style were similar for *A. oreoscordum* and *A.oreoprasoides* (sect. *Nigrimontana*) and differentiated them from two species of sect. *Companulata*, *A. inconspicuum* and *A. barszcewskii* (Table 1).

### Phylogenetic study of *Allium* species

Four different datasets were generated in this study. First two datasets were related to ITS sequences of local species and sequences for worldwide species collected from the NCBI database, respectively. Next two datasets were related to *mat*K sequences for local and worldwide accessions from the NCBI database, respectively. The length of ITS in local accessions varied from 616 bp (*A. eriocoleum*) to 638 bp (*A. fistulosum*, *A. altaicum*, Additional file 2), and the alignment was adjusted by introducing gaps using MEGA 5.0. This resulted in 671 bp as a total alignment length. The number of polymorphic nucleotides was 415, or 61.8% out of the total number of nucleotides (Additional file 2). The length of *mat*K was more conservative and varied from 779 bp in *A. caesium* and *A. sabulosum* to 788 bp in all remaining species (Additional file 3). The number of polymorphic sites in *mat*K for all local species was 73, or 9.3% out of total number of nucleotides (Additional file 3). Therefore, in this study the variability of ITS was in 6.6 times higher than *mat*K.

### Genetic analysis of local *Allium* species using ITS (dataset 1)

**Fig. 2:**
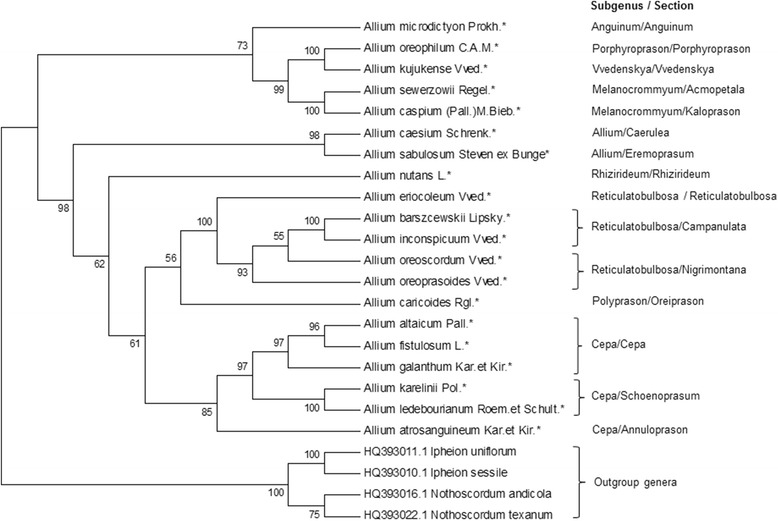
Neighbor-Joining phylogenetic tree resulted from analysis of the ITS sequences of twenty local *Allium* species and four outgroup taxa. The subgenera and sectional classification are given according to Friesen et al. [8]. The length of branches is based on Maximum Composite Likelihood and numbers at nodes shows a probability bootstrap. * is indication of local species and taxa with reference numbers representing accessions from the NCBI database

In total 20 collected species from Kazakhstan listed in Additional file 1 were analyzed using ITS sequences. The Neighbor-Joining phylogenetic tree constructed by using four outgroup species separated 20 species in two distinct clades. Figure 2 provides relationships of these *Allium* species.

The first clade included species from subgenera *Anguinum, Porphyroprason, Vvedenskaya*, and *Melanocronium,* which are part of the second evolutionary line. The second clade included species that part of third evolutionary line. Species in subgenus *Cepa* formed a sister subclade with other subclade consisting from *A. caricoides* and species in subgenus of *Reticulatobulbosa.* The species in the subgenus *Reticulatobulbosa* have formed a separate group within the second clade, and the *A. oreoprasoides* was the closest taxon to the *A. oreoscordum*.

### Genetic analysis of s set of worldwide occurring *Allium* species using ITS dataset (dataset 2)

**Fig. 3:**
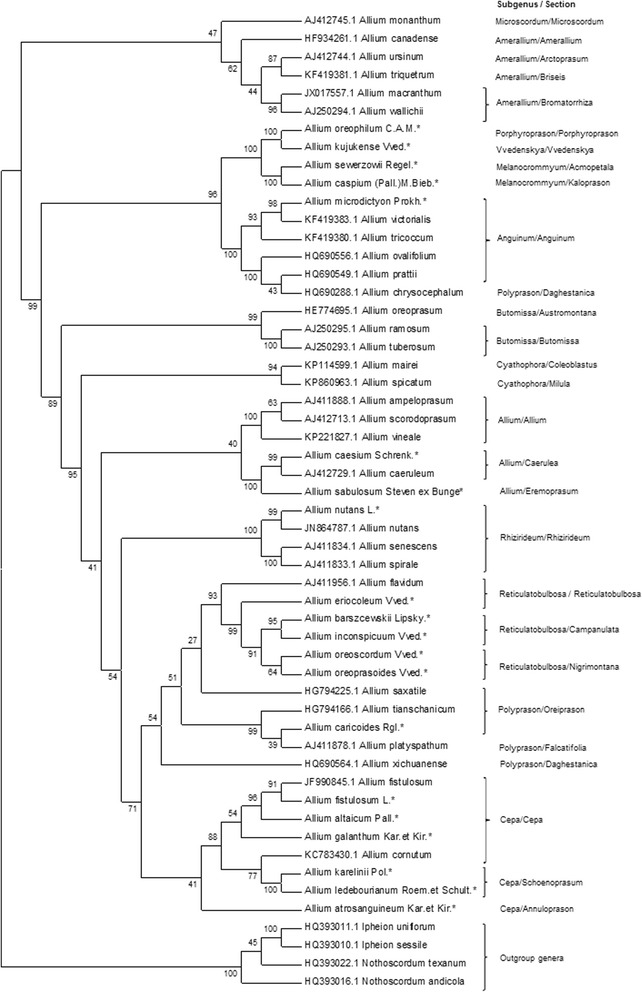
Neighbor-Joining phylogenetic tree resulted from analysis of the ITS sequences of twenty local, thirty worldwide *Allium* species and four outgroup taxa. The subgenera and section classification are given according to Friesen et al. [8]. The length of branches is based on Maximum Composite Likelihood and numbers at nodes shows a probability bootstrap. * is indication of local species and taxa with reference numbers representing accessions from the NCBI database

The dataset 2 included ITS sequences of 50 *Allium* species, consisting of 20 local and 30 worldwide distributed species available at the NCBI database. (Fig. 3).

The generated phylogenetic tree was in congruence with results from the first dataset and supported the theory of a monophyletic *Allium* origin. The only misplaced species in the phylogenetic tree in this study was *A. chrysocephalum* (subg. *Polyprason*, sect. *Daghestanica*), which was grouped together with species from subg. *Anguinum*. The topology of the tree is suggesting that species in subg. *Reticulatobulbosa* derived from species in subgenus *Polyprason* (Fig. 3) and formed a subclade, which is a sister subclade to species in subg. *Cepa*. Within *Reticulatobulbosa*, similarly to the results from the analysis of the dataset 1, *A. inconspicuum* and *A. barszchewskii* formed a sister group, and *A. oreoprasoides* and *A. oreoscordum* formed a second one. The bootstrap support was 91%.

### Genetic analysis of local *Allium* species using *mat*K dataset (dataset 3)

**Fig. 4:**
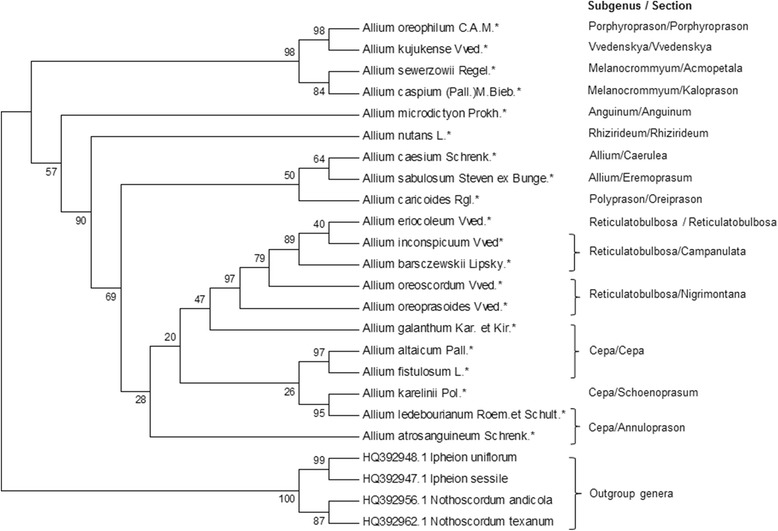
Neighbor-Joining phylogenetic tree resulted from analysis of the *mat*K sequences of twenty local *Allium* species and four outgroup taxa. The subgenera and sectional classification are given according to Friesen et al. [8]. The length of branches is based on Maximum Composite Likelihood and numbers at nodes shows bootstrap support values. * is indication of local species and taxa with reference numbers representing accessions from the NCBI database

The study of dataset 3 was based on the analysis of sequences from 20 local *Allium* species of plastid genome marker *matK* (Fig. 4).

The Neighbor-Joining dendrogram generated three distinct clades with the first clade containing species from subgenera *Melanocronium, Vvedenskaya,* and *Porphyroprason*; the second clade with species in subgenera *Allium* and *Polyprason*; and the third clade with species of subgenera *Cepa* and *Reticulatobulbosa*. In the topology of the tree *A. microdictyon* and *A. nutans* showed intermediate positions between groups of the first and the second evolutionary lines, although the former species was closer to the first evolutionary lines and latter to the second line. As in the ITS tree, the results within *Reticulatobulbosa* suggested close genetic relationship between *A. oreoprasoides* and *A. oreoscordum.*

### Genetic analysis of worldwide occurring *Allium* species using *mat*K dataset (dataset 4)

**Fig. 5:**
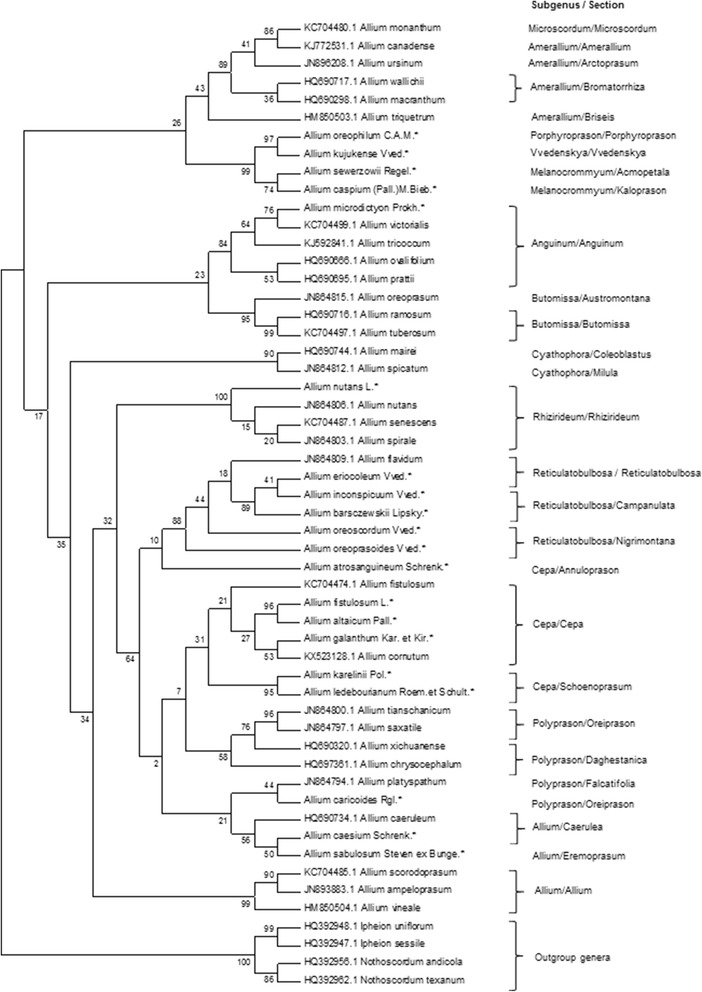
Neighbor-Joining phylogenetic tree resulted from analysis of the *mat*K sequences of twenty local and thirty worldwide occurring *Allium* species and four outgroup taxa. The subgenera and sectional classification are given according to Friesen et al. [8]. The length of branches is based on Maximum Composite Likelihood and numbers at nodes shows bootstrap support values (%). * indicates local species and taxa with reference numbers representing accessions from the NCBI database

As in the ITS study, the generated *mat*K phylogenetic tree supported the theory of a monophyletic *Allium* origin. However, unlike the study of dataset 2, the analysis of 50 accessions using *matK* has not confirmed the topology of *Allium* groups based on three generated clades (Fig. 5) containing species attributed to the three evolutionary lines.

The first difference was that species in subgenera *Melanocronium, Vvedenskaya,* and *Porphyroprason* (second evolutionary line) were grouped together with subgenera *Amerallium* and *Microscordum* (first evolutionary line). The second difference in the phylogenetic tree was the grouping of species in subgen. *Butomissa* (third evolutionary line) together with species in *Anguinum* (second evolutionary line) in the same clade. However, *A. oreoprasoides* and *A. oreoscordum* in subgenus *Reticulatobulbosa* were again positioned together with bootstrap support of 88% (Fig. 5).

## Discussion

Although the variability of *mat*K in *Allium* species is smaller than in ITS, the phylogenetic trees using markers from two different genomes confirmed earlier findings of monophyletic origin in *Allium* genus [8, 14]. Our data suggested that the collected 20 local *Allium* species belong to subgenera in the second and third evolutionary lines postulated by Friesen et al. [8]. The notable difference between the results from datasets 1 and 3 is that unlike in ITS, the dendrogram in the *mat*K analysis is suggesting that species in subgen. *Cepa* evolved earlier than those in subgen. *Reticulatobulbosa*, and these two subgenera have a common ancestral node (Fig. 4). Similar results can be seen from the observations of phylogenetic trees from datasets 2 and 4, which include larger numbers of taxa added from the NCBI database. The analysis of dataset 4 based on *mat*K sequences (Fig. 5) is suggesting that species in *Reticulatobulbosa* evolved from species in subgen. *Cepa*. This result is not in congruence with the recent classification [8] of *Allium*, and most probably can be explained by lower variability in *mat*K. Therefore, most important output can be observed from the ITS topology of phylogenetic tree for study of worldwide species (dataset 2). Unlike in phylogenetic tree generated in [8], where subclades *Polyprason*, *Reticulatobulbosa* and *Cepa* formed three sister subclades, in this study only two subclades were detected. The first subclade was species from subgenera *Cepa*, while second one consisted from species of *Polyprason* and *Reticulatobulbosa*. The other misplacement of species in the worldwide ITS phylogenetic tree was a grouping of *A. chrysocephalum* (subgenus *Polyprason*, section *Daghestanica*) together with species from subgenus *Anguinum,* which may potentially happened due to inaccuracies of sequences in the database.

In this study five endemic and rare species within subgenus *Reticulatobulbosa*, representing sections *Reticulatobulbosa, Companulata,* and *Nigrimontana* were analyzed. All five species were collected in Karatau Mountains (black mountains in translation from Kazakh) in South Kazakhstan region. The name of the section *Nigrimontana* is coinciding with the name of these mountains, and most probably related to this place and reflects richness and uniqueness of the local flora, including in possessing of many wildly growing onion species [24]. The phylogeny of both ITS and *matK* is suggesting that within subgenus three sub groups can separated. The representative of the first group is *A. eriocoleum* (section *Reticulatobulbosa*) was positioned mostly apart from the remaining four species (Figs. 2, 3, 5). In all four datasets, the *A. oreoprasoides* (section *Nigrimontana*) and *A. oreoscordum* formed second sub group, while *A. inconspicuum* and *A. barszchewskii* (both species from section *Companulata)* formed third sub group. Based on these findings the *A. oreoscordum* with high probability can be positioned in the section *Nigrimontana*. Morphological description of species from sections *Nigrimontana* and *Companulata* (Table 1) can be used as an additional confirmation for this assumption.

The result of this study was an initial effort in large scale project oriented in thorough description of endemic and rare species in Kazakhstan based on DNA barcoding approach. It was determined that in the analysis of 20 local *Allium* accessions the level of variability in ITS was 6.6 higher than in *matK*. The other important result was an assessment of taxonomic statuses of endemic and rare *Allium* species growing in Kazakhstan. The study confirms monophyletic origin of *Allium* genus that was established in several published reports [8, 13, 14]. In addition, a detailed study on comparison of *Reticulatobulbosa* species was performed with a major attention to morphological and molecular genetic description of species grown in Karatau State Natural Reserve.

## Conclusions

Despite fundamental study on phylogenetic taxonomy of *Allium* genus by Friesen and co-authors [8], there are still many poorly described *Allium* taxa available in different parts of the World. In this study, in order to assess the phylogeny of 20 endemic, rare and economically important *Allium* species from Kazakhstan, two DNA barcoding markers ITS and *matK* were applied. The obtained results suggested that the variability of ITS sequences in studied 20 *Allium* species was in 6.6 times higher than in *matK*. Generated phylogenetic trees using ITS sequences were well in congruence with existing new phylogenetic classification (Friesen et al., 2006) and confirmed monophyletic origin of the genus. Both ITS and *matK* analyses suggested that regional endemic *A. oreoscordum* with high probability can be positioned in the section *Nigrimontana* of the subgenus *Reticulatobulbosa*. This result was also confirmed by the assessment of morphological traits for four different *Allium* species in the subgenus *Reticulatobulbosa.* The study is another contribution to taxonomy clarification in *Allium*.

## Methods

### Plant material

Collecting trips were organized in 2015 and 2016 in five different regions of the country (Fig. 1) and resulted in sampling of 20 different endemic, rare and economically important *Allium* species.

The locations and geographic characterizations of the collecting sites are provided in the table (Additional file 1). Voucher specimens were deposited in the herbarium of the Institute of Plant Biology and Biotechnology (Kazakhstan). From five to ten plants were described for each species in every collecting site (Table 1). The elevation from above sea level is ranged from 96 to 2669 m. Leaf samples of plants growing distantly apart were collected in plastic bags containing silica gel for DNA extraction.

### DNA extraction, amplification and sequencing.

Total genomic DNA was extracted from dry or fresh leaves according to the Dellaporta DNA extraction protocol [25]. PCR fragments were amplified from the maturase K gene in chloroplast genome (*matK*) and the nuclear ribosomal complex including the internal transcribed spacers 1 and 2, and 5.8S rRNA.

All PCR reactions were performed in total 16 μl volumes in Veriti Thermo cycler (Applied Biosystems, Foster City, CA, USA). Nucleotides sequences of ITS [26] and *matK* [20] primers and sizes of amplicons are represented in Table 2.

**Table 2:** Primers for ITS1–5.8S–ITS2 region and *matK* gene

Primers	Nucleotide sequence	Annealing temperature	Amplicon sizes
ITS1nF ITS4nR	5’-AGAAGTCGTAACAAGGTTTCCGTAGG- 3′ 5’-TCCTCCGCTTATTGATATGC- 3’	58 °C	638 bp
*matK*-F *matK*-R	5’-CCTATCCATCTGGAAATCTTAG- 3′ 5’-GTTCTAGCACAAGAAAGTCG- 3’	50 °C	788 bp

Whole volume of each PCR products was checked by electrophoresis in 1.5% agarose gel at 80 V voltage for 40 min. Single bands with expected sizes for *matK* and ITS were visualized, cuted out from gel and purified using ULTRAPrep® Agarose Gel Extraction Mini Prep Kit (AHN Biotechnologie GmbH, Nordhausen, Germany) according to the protocol provided by the company. Purified DNA amplicons were used for the sequence reactions with forward and reverse primers separately. All reactions were performed with the BigDye Terminator Cycle Sequencing technology (Applied Biosystems, Foster City, CA, USA) according to protocols of the company.

### Alignment and phylogenetic analyses

Generated sequences of local *Allium* samples were imported in MEGA 5 software [27] and aligned by using ClustalW program [28]. In addition, the sequences for ITS and *matK* of local species were aligned with sequences of *Allium* species from the NCBI reference database [29]. For the construction of phylogenetic tree the Maximum Composite Likelihood model [30], Neighbor-Joining statistical method [31], and the 1000 replication bootstrap test were used.

## Additional files

Additional file 1: The list of *Allium* species collected in five regions of Kazakhstan (2015–2016). Endemic species were highlighted in bold. (PDF 312 kb)

Additional file 2: Polymorphic sites of ITS in twenty *Allium* species collected in Kazakhstan. (XLSX 44 kb)

Additional file 3: Polymorphic sites of *matK* in twenty *Allium* species collected in Kazakhstan. (XLSX 15 kb)
