# Supplementary material for: Taxonomic assessment of Allium species from Kazakhstan based on ITS and matK markers
Source: BMC Plant Biol. 2017 Dec 28;17(Suppl 2):258. doi: 10.1186/s12870-017-1194-0 (PMC5751797; doi:10.1186/s12870-017-1194-0)
Supplement: Supplementary file 1 — The list of Allium species collected in five regions of Kazakhstan (2015–2016). Endemic species were highlighted in bold. (PDF 312 kb) [file 12870_2017_1194_MOESM1_ESM.pdf]

**Subgenus/Section (Friesen et al., 2006)**	**Species**	**Collecting Regions**	**Coord. (N)**	**Coord. (E)**	**Altitude a.s.l., (m)**	**ITS length (bp)**	***matK* length (bp)**
Anguinum/Anguinum	*A. microdictyon* Prokh.	East Kazakhstan (Ridder region, Seryy lug settlement)	50^{0} 21' 27"	83^{0} 53' 42"	1169	637	788
Porphyroprason/Porphyroprason	*A. oreophilum* C. A. Mey.	South Kazakhstan (Gorge Saryaygyr, Mountain Ular)	42^{0}06′ 54.0"	70^{0}18′ 34.0"	2605	628	788
Vvedenskya/Vvedenskya	***A. kujukense* Vved.**	South Kazakhstan (Kazanbulak, Gorge Kabylandysay)	43°41′ 57.6”	68°47′ 43,5”	1034	626	788
Melanocrommyum/Acmopetala	*A.sewerzowii* Regel	South Kazakhstan (Tolebi region, Ogem village, Ugam range, Aktas Gorge)	41^{0}53′ 19.4"	70°05′ 55.4"	1990	636	788
Melanocrommyum/Kaloprason	*A. caspium* (Pall.) M. Bieb.	West Kazakhstan (Tuyesu Sands)	43^{0} 21' 427"	53^{0} 32' 934"	96,2	625	788
Rhizirideum/Rhizirideum	*A. nutans* L.	East Kazakhstan (mountain area Akimbay)	49°11.272'	86°12.566'	1242	637	788
Polyprason /Oreiprason	***A. caricoides* Rgl.**	South Kazakhstan (Gorge Saryaygyr)	42° 07 ′33.2"	70° 17 ′59.8"	2669	635	788
Reticulatobulbosa/Reticulatobulbosa	*A.eriocoleum* Vved.	South Kazakhstan (Karatau, Sualma)	43° 41 ′29.2"	68° 33 ′13.2"	755	616	788
Reticulatobulbosa/ Campanulata	***A.barsczewskii* Lipsky**	South Kazakhstan (Gorge Saryaygyr)	42° 07 ′05.2"	70° 18 ′40.0"	2428	628	788
Reticulatobulbosa/ Campanulata	*A.inconspicuum*	South Kazakhstan (Karatau, Sualma)	43° 45 ′08.6"	68° 32 ′06.7"	1091	628	788
Reticulatobulbosa/Nigrimontana	*A. oreoscordum* Vved.	South Kazakhstan (Kazanbulak, Gorge Kabylandysay)	43°42′ 24.9”	68°46′ 56,6”	940	629	788
Reticulatobulbosa/Nigrimontana	***A. oreoprasoides* Vved.**	South Kazakhstan (Kazanbulak,	43°41′ 57.6”	68°47′ 43,5”	1034	629	788

		Gorge Kabylandysay					
Cepa/Cepa	*A. galanthum* Kar. et Kir.	Central Kazakhstan (Bektauata)	47°23' 147"	74°50' 210"	594	636	788
Cepa/Cepa	*A. fistulosum* L.	East Kazakhstan (Ridder region, surroundings of Ulba village	50° 15′ 01″	83° 20′ 06″	625	638	788
Cepa/Cepa	*A. altaicum* Pall.	East Kazakhstan (Katon-Karagai region, South Altai Tarbagatai range)	49° 05′ 41″	86° 12′ 35″	1950	638	788
Cepa/Schoenoprasum	***A. karelinii* Poljak.**	South-east Kazakhstan (Almaty State Nature Reserve)	43^0 13' 31.2"	77^0 17' 28.4"	1634	627	788
Cepa/Schoenoprasum	***A. ledebourianum*** Schult. et Schult. f.	East Kazakhstan (Ridder region, Seriy lug settlement)	50^0 21' 27"	83^0 53' 54"	1170	626	788
Cepa/Annuloprason	*A. atrosanguineum* Kar. et Kir.	South-east Kazakhstan (Almaty State Nature Reserve)	43^0 12' 23.6"	77^0 20' 32.5"	2325	636	788
Allium/Caerulea	*A. caesium* Schrenk	South Kazakhstan (Karatau, Sualma)	43^{0}41′ 29.2"	68^{0}33′ 13.2"	755	626	779
Allium/ Eremoprasum	*A. sabulosum* Stev.	South-east (Right bank of the Ili river, in the area of the Kapchagai reservoir, sandy places)	43^{0}19′ 41.37″	75^0 06′ 20.27″	1100	637	779
